# Quantification of the zymogenicity and the substrate-induced activity enhancement of complement factor D

**DOI:** 10.3389/fimmu.2023.1197023

**Published:** 2023-05-22

**Authors:** Ráhel Dani, Gábor Oroszlán, Róbert Martinusz, Bence Farkas, Bernadett Dobos, Evelin Vadas, Péter Závodszky, Péter Gál, József Dobó

**Affiliations:** Institute of Enzymology, Research Centre for Natural Sciences, Budapest, Hungary

**Keywords:** innate immunity, complement, serine protease, proenzyme, kinetics, factor D

## Abstract

Complement factor D (FD) is a serine protease present predominantly in the active form in circulation. It is synthesized as a zymogen (pro-FD), but it is continuously converted to FD by circulating active MASP-3. FD is a unique, self-inhibited protease. It has an extremely low activity toward free factor B (FB), while it is a highly efficient enzyme toward FB complexed with C3b (C3bB). The structural basis of this phenomenon is known; however, the rate enhancement was not yet quantified. It has also been unknown whether pro-FD has any enzymatic activity. In this study, we aimed to measure the activity of human FD and pro-FD toward uncomplexed FB and C3bB in order to quantitatively characterize the substrate-induced activity enhancement and zymogenicity of FD. Pro-FD was stabilized in the proenzyme form by replacing Arg^25^ (precursor numbering) with Gln (pro-FD-R/Q). Activated MASP-1 and MASP-3 catalytic fragments were also included in the study for comparison. We found that the complex formation with C3b enhanced the cleavage rate of FB by FD approximately 20 million-fold. C3bB was also a better substrate for MASP-1, approximately 100-fold, than free FB, showing that binding to C3b renders the scissile Arg-Lys bond in FB to become more accessible for proteolysis. Though easily measurable, this cleavage by MASP-1 is not relevant physiologically. Our approach provides quantitative data for the two-step mechanism characterized by the enhanced susceptibility of FB for cleavage upon complex formation with C3b and the substrate-induced activity enhancement of FD upon its binding to C3bB. Earlier MASP-3 was also implicated as a potential FB activator; however, MASP-3 does not cleave C3bB (or FB) at an appreciable rate. Finally, pro-FD cleaves C3bB at a rate that could be physiologically significant. The zymogenicity of FD is approximately 800, i.e., the cleavage rate of C3bB by pro-FD-R/Q was found to be approximately 800-fold lower than that by FD. Moreover, pro-FD-R/Q at approximately 50-fold of the physiological FD concentration could restore half-maximal AP activity of FD-depleted human serum on zymosan. The observed zymogen activity of pro-FD might be relevant in MASP-3 deficiency cases or during therapeutic MASP-3 inhibition.

## Introduction

The complement system is an essential part of innate immunity representing a primary line of defense against pathogenic microorganisms and malfunctioning self-cells. It is composed of ca. 40 soluble and surface-bound proteins (proteolytic enzymes, non-enzymatic effectors, regulators, and receptors), which are organized into a complex network. The complement system can be activated *via* three traditionally distinct routes: the classical (CP), the lectin (LP), and the alternative pathways (AP). The three pathways merge at the cleavage of the C3 component and continue in a common effector route, the terminal pathway (1–3). The AP provides an amplification loop regardless of the initial activation route; therefore, the AP C3 convertase formation is a central event of complement activation in general. The AP C3 convertase is generated from the AP pro-convertase, that is complement factor B (FB) complexed with C3b (C3bB). Factor D (FD) cleaves the FB component of this complex giving rise to the mature AP C3 convertase, C3bBb. C3bBb has a short lifetime and dissociates rapidly on its own (4); however, it can be stabilized by properdin or destabilized by certain factors, including factor H (FH), and degraded by factor I (FI) in the presence of co-factors, like FH (5). The AP can also be initiated spontaneously *via* the so-called tick-over mechanism (6–8). The C3 component hydrolyzes in the solution phase at a low rate. The emerging C3(H_2_O) has a C3b-like conformation. FB binds to C3(H_2_O) and it is cleaved by FD. The resulting C3(H_2_O)Bb is a fluid-phase C3 convertase that can cleave C3. If this cleavage occurs near a surface, C3b can deposit and initiate the AP amplification loop. Self-surfaces are protected against spontaneous AP activation by membrane-associated inhibitors.

FD is an essential enzyme for AP activation. It circulates predominantly in an active-like mature form; nevertheless, its activity is greatly limited due to the presence of a self-inhibitory loop (9). A key residue in this loop is Arg^227^ (m202, c218), which forms a salt bridge with Asp^202^ (m177, c189) at the bottom of the S1 substrate-binding pocket. As a result, distortion of the catalytic triad can be observed in the structure of mature FD (9, 10). Note that the numbers by default refer to the protein precursor as found in the UniProt database, and numbers in parenthesis, when relevant, refer to the mature enzyme (m) or the traditional chymotrypsin (c) numbering for easier identification. Upon binding to its natural substrate C3bB, the self-inhibitory loop is displaced and the catalytic triad of FD becomes properly aligned to cleave the scissile Arg^259^-Lys^260^ (m234-235) bond of FB. This arrangement was observed in the structure of the FD-S/A-FB-C3b complex, formed between the AP C3 pro-convertase and the catalytically inactive Ser^208^ (m183, c195) to the Ala variant of FD (FD-S/A) (11). In this complex, FB is in a cleavage-prone “open” conformation, while FD has a conformation typical for active, trypsin-like serine proteases (except for the replacement of the active site Ser to Ala). C3b-complexed FB was also observed in two conformations, the so-called “open” and “closed” by electron microscopy (12). These conformations seem to be in equilibrium in solution favoring the cleavage-prone open conformation. Properdin can bind to and stabilize the AP C3bB pro-convertase and probably binds to the “open” conformation of this complex (13). Uncomplexed free FB has the scissile Arg^259^-Lys^260^ (m234-235) bond in a (“closed”) conformation that is not accessible for cleavage (14). Based on these observations, a dual mechanism can be drawn. Upon complex formation with C3b, FB attains a cleavage-susceptible “open” conformation, and binding of FD to the “open” conformation of FB increases the activity of FD by a substrate-induced conformational rearrangement.

The cleavage rate of C3(H_2_O)B by FD had been measured earlier (15); however, the cleavage rate of free FB by FD has not been quantified. This reaction is presumably very slow to the extent that FB has been considered not to be cleavable by FD. Nevertheless, we attempted to quantify this very slow reaction. MBL-associated serine protease-1 (MASP-1) was also included in this study as a protease with relatively high activity and broad specificity (16). Using this enzyme, we attempted to quantify the rate enhancement due to the increased susceptibility of FB upon complex formation with C3b.

For a long time, FD had been assumed to be secreted into the blood as a mature, “active” protease, because after isolation from blood samples, always the mature form was obtained (17, 18). Now, there is a consensus that initially zymogen (proenzymic) FD (pro-FD) is secreted into the blood (19, 20), which is continuously activated by the active form of MBL-associated serine protease-3 (MASP-3) (19, 21, 22). MASP-3 and MASP-1 are two splice products of the same *MASP1* gene. These two enzymes are traditionally considered as components of the LP (23–25). The blood of *MASP1* knockout (KO) mice contains pro-FD; however, initially, it was uncertain which one of the two proteases, if any, is responsible for FD maturation (26–28). Later, it was proven in human blood samples using an MASP-3-specific inhibitor, and in mice deficient for MASP-3 only, that in both species MASP-3 is the physiological activator of pro-FD (21, 22), whereas a proprotein convertase, PCSK6 (proprotein convertase subtilisin/kexin-6), was found to activate MASP-3 in human blood (29). Nevertheless, the human serum of a 3MC (Malpuech–Michels–Mingarelli–Carnevale) syndrome patient deficient for both MASP-1 and MASP-3 had residual AP activity, which indicated that a backup mechanism might exist in the serum containing predominantly pro-FD (28). Ruseva et al. (30) also showed that in KO mice, deficient for both FH (*FH^−/−^*) and MASP-1/3 (*MASP1^−/−^*), the AP is continuously active characterized by the depletion of intact FB. These observations indicate that pro-FD might be cleaved by another protease with low efficiency, or pro-FD might have some activity, or possibly the two effects occur simultaneously.

In this study, we had two major goals. First, we wanted to quantify the substrate-induced activity enhancement of FD by measuring its activity on free FB and C3bB as substrates. MASP-1 was included in the study in order to quantify the extent of the increased susceptibility of FB for cleavage upon complex formation with C3b. MASP-3, however, was included because it was shown earlier by others that it might be a direct FB activator, using C3bB as a substrate (27). Second, we wanted to clarify if pro-FD has any enzymatic activity and quantified it on the same substrates. A major finding was that pro-FD possesses significant activity toward C3bB, which can provide a possible explanation for the abovementioned observations.

## Materials and methods

### MASP-1, MASP-3, reagents, FB, serum FD, and C3b

Recombinant human MASP-1 catalytic fragment (cf) and MASP-3cf were expressed, refolded, and purified as described previously (16, 31, 32). The catalytic fragment represents the last three (CCP1–CCP2–SP) domains of both enzymes. MASP-1cf is produced as an active enzyme, while MASP-3cf is initially produced as a zymogen, which is activated *in vitro* by MASP-1cf, and further purified as described to remove MASP-1cf (33). The purified active MASP-3cf sample contained approximately 10% uncleaved zymogen MASP-3cf, due to incomplete cleavage by MASP-1cf and because it co-eluted with the active counterpart during purification after activation. On the other hand, zymogen MASP-3cf does not have any measurable proteolytic activity (33). Commercial reagents were purchased from Merck (Darmstadt, Germany, Sigma-Aldrich brand). Human, serum-derived, purified FD and FB were purchased from Merck (Darmstadt, Germany, Calbiochem brand).

C3b was prepared from human EDTA plasma as described previously (34), then it was further purified by cation-exchange chromatography, as follows. C3b was applied to a 1-ml YMC-BioPro SP-F column (YMC Co. Ltd., Kyoto, Japan) in 10 mM of MES pH 5.8 and eluted in a 20-column volume (CV) linear NaCl gradient (0–300 mM of NaCl). The buffer of the eluted C3b was exchanged to 100 mM of NaCl, 50 mM of Tris pH 7.4, and 5 mM of MgCl_2_ buffer by dialysis, and then it was concentrated to approximately 2 mg/ml (approximately 11 μM) on PES 10-kDa cutoff spin concentrators (Thermo Fisher, Rockford, IL, USA, Pierce brand).

The molecular weights and extinction coefficients are as follows: 45.5 kDa, 69,870 M^−1^ cm^−1^ (1.54 ml mg^−1^ cm^−1^) for MASP-1cf; 48.1 kDa, 81,205 M^−1^ cm^−1^ (1.69 ml mg^−1^ cm^−1^) for MASP-3cf; ~93 kDa, 122,420 M^−1^ cm^−1^ (1.32 ml mg^−1^ cm^−1^) for FB; 24.4 kDa, 28,460 M^−1^ cm^−1^ (1.17 ml mg^−1^ cm^−1^) for FD; and ~180 kDa, 176,700 M^−1^ cm^−1^ (0.98 ml mg^−1^ cm^−1^) for C3b. The molar extinction coefficients and molecular weights of the non-glycosylated proteins were calculated from the protein sequences using the Expasy ProtParam tool. The approximate molecular weights of the glycoproteins (FB and C3b) isolated from human serum or plasma are based on published data (35, 36). UniProt identifiers of the human proteins are P48740-1 for MASP-1, P48740-2 for MASP-3, P00746 for FD, P00751 for FB, and P01024 for C3. Bovine chymotrypsinogen A (UniProt P00766) was used as a template for chymotrypsin numbering, when applicable.

### Expression and purification of recombinant human FD

Earlier, C-terminally His_6_-tagged pro-FD (pro-FD-H_6_) and mature FD (FD-H_6_) were produced and purified as described (33). For this study, we constructed a tag-free recombinant human pro-FD, which was produced in the same expression system, the baculovirus insect cell system, as pro-FD-H_6_. The segment encoding the His_6_-tag was removed from the original DNA construct (33) by mutagenesis introducing a stop codon (underlined) and an *Avr*II (CCTAGG) restriction site using the 5′-CCTGGATCGACAGCGTCCTGGCCTAGGATCATCATCATCATTAAGAATTCTAGAAGG-3′ primer and the QuikChange Multi Site-Directed Mutagenesis Kit (Agilent Technologies, Santa Clara, CA, USA). The purification strategy was modified due to the lack of a tag. As a first step, low molecular weight media components were removed on a Sephadex G-25 (GE Healthcare, now Cytiva, Marlborough, MA, USA) column. Pro-FD in 20 mM of NaH_2_PO_4_ pH 6.8 buffer was applied first onto a 10-mm × 100-mm YMC BioPro S30 (YMC Co. Ltd., Kyoto, Japan) column, then onto a 10-mm × 100-mm SP Sepharose HP (GE Healthcare) column. Pro-FD was eluted using a 12-CV 0–350 mM of NaCl gradient in the same buffer in both cases. Fractions containing pro-FD were combined, activated by trace amounts of trypsin, and then purified on an SP Sepharose HP column to completely remove trypsin as previously described (33). Since human FD does not have any glycosylation sites, the tag-free recombinant human FD is virtually identical to the blood-derived enzyme.

### Recombinant, stable proenzymic R25Q variant of human pro-FD (pro-FD-R/Q)

Recombinant human pro-FD-R/Q DNA was produced from the wild-type construct (see above) by mutagenesis using the 5′-GCCGCCCCGTGGTCAGATCCTGGGCGGC-3′ primer and the QuikChange Multi Site-Directed Mutagenesis Kit (Agilent Technologies, Santa Clara, CA, USA). The altered codon is underlined. In the protein product, Arg^25^ (c15) of the activation site is replaced by Gln. Expression and purification were carried out as described above for wild-type pro-FD. After removal of the signal peptide within the cells (segments 1–19), the stable proenzymic pro-FD-R/Q mutant is expected to carry the APPRGQ propeptide at the N-terminus as opposed to an APPRGR propeptide (segments 20–25) found in the wild-type proenzyme (33, 37). The molecular weight and extinction coefficient are 25.0 kDa and 28,460 M^−1^ cm^−1^ (1.14 ml mg^−1^ cm^−1^) for pro-FD-R/Q.

### Cleavage assays

Cleavage assays were performed in 100 mM of NaCl, 50 mM of Tris pH 7.4, and 5 mM of MgCl_2_ (assay buffer) at 37°C. Free FB substrate was used at 1.1 μM final concentration. The complex substrate C3bB was prepared from FB and C3b at 1.1 and 1.3 μM final concentrations, respectively. A slight excess of C3b was used to make sure there is sufficient C3b available for every FB molecule. The complex substrate in this paper will be referred to as 1.1 μM of C3bB; however, it must be considered that its components are just partially associated, and the K_D_ is approximately 1.4 μM (38). Background cleavage of the substrates was checked without the added cleaving enzymes. The cleaving enzymes, recombinant FD, pro-FD-R/Q, MASP-1cf, and MASP-3cf were used at the final concentrations listed in **Table 1**. Samples were withdrawn periodically as listed in **Table 1**, and the reactions were stopped by adding an equal volume of reducing sample buffer (125 mM of Tris pH 6.8, 10 V/V% of glycerol, 5 m/V% SDS, 0.02% bromophenol blue) and immediate heating at 95°C for 2 min. The samples were separated by SDS-PAGE under reducing conditions, and the gels were stained with Coomassie Brilliant Blue G-250. The gels were scanned by an Epson Perfection V850 Pro Scanner in transparent mode, and densitometry was carried out with the ImageJ software (version 1.52a, National Institute of Health, USA).

**Table 1:** Composition and sampling frequency of the cleavage reaction mixtures.

Enzyme	Enzyme concentration (nM)	Substrate (1.1 µM)	Typical sampling for densitometry
None (background)	–	C3bB	0, 2, 4, 6 h, 1 day
	–	FB	0, 1, 2, 3, 5, 7 days
FD	0.25	C3bB	0, 5, 10, 15, 20, 30, 60, 180 min
	2,000	FB	0, 1, 2, 3, 5, 7 days
pro-FD-R/Q	100	C3bB	0, 5, 10, 15, 20, 25, 30, 60, 180 min
	2,000	FB	0, 1, 2, 3, 5, 7 days
MASP-1cf	1,000	C3bB	0, 15, 30, 45, 60, 90, 120, 180 min
	2,600	FB	0, 2, 4, 6, 8 h, 1 day
MASP-3cf	2,000	C3bB	0, 1, 2, 4, 6 h, 1 day
	10,000	FB	0, 1, 2, 4, 6 h, 1 day

A maximal cleavage reaction mixture was prepared with FD (0.25 nM) and C3bB (1.1 μM) and at least 3 h of incubation. This sample was applied to the gels representing low-efficiency reactions. This was necessary for fitting purposes.

Quantification of the cleavage product Bb gave the best fitting for all reactions. This is especially true for low-efficiency reactions where the decrease in the intensity of the substrate (FB) is minimal. The observed first-order rate constants (*k*_obs_) were determined by non-linear regression using the *I*_P_ = *I*_B_ + *I*_max_·× [1 − exp(−*k*_obs_ × *t*)] equation and the OriginPro 8 software (OriginLab, Northampton, MA, USA), where *I*_P_ is the intensity of the product (Bb) band, *I*_B_ is the intensity of the gel background, and *I*_max_ is the fitted or the hypothetical maximal intensity of the fully cleaved product. In the case of the low-efficiency reactions, the *I*_max_ intensity was derived from the maximal cleavage reaction mixture (see above), whereas when the reaction was nearly complete, it was a fitted parameter. For figure preparations, the background intensity was subtracted from the raw data, and the background-corrected values were normalized to the intensity of the maximal cleavage and plotted as percentage values using the OriginPro 8 software.

In order to compare the different reactions, *k*_obs_/[*E*]_T_ values were calculated, where [*E*]_T_ is the total enzyme concentration. The *k*_obs_/[*E*]_T_ value can be considered as an approximation of the catalytic efficiency (*k*_cat_/*K*_M_), and according to the Michaelis–Menten kinetics, the *k*_obs_/[*E*]_T_ value is equal to the *k*_cat_/*K*_M_, if the substrate concentration is much less than the *K*_M_.

For curiosity, we have determined the cleavage rates of C3bB for recombinant FD-H_6_ and serum-derived FD. Recombinant FD-H_6_ and recombinant FD had virtually the same activity, while serum-derived FD was slightly less active probably due to lower purity (data not shown). Also, for curiosity, we measured the activities of all enzymes in the assay buffer supplemented with 5 mM of CaCl_2_, with virtually identical results (data not shown).

### Human serum and plasma

Blood was drawn from 10 healthy donors and collected into S-Monovette (Sarstedt, Nümbrecht, Germany) tubes prepared with a clotting activator (silicate) or K_3_-EDTA. Serum samples and plasma samples were combined separately and stored at −80°C in aliquots. Blood collection was conducted in conformity with the World Medical Association Declaration of Helsinki and also approved by the local ethics committee (permission number: TUKEB 9190-1/2017/EKU). Informed consent was obtained from the donors. No personally identifiable information was obtained during this study.

### Alternative pathway activity measurement by ELISA

High-binding microtiter plates (Greiner Bio-One, Kremsmünster, Upper Austria) were coated with 100 µg/ml of zymosan (Santa Cruz Biotechnology, Dallas, TX, USA, cat. no. sc-258367A) in 15 mM of Na_2_CO_3_, 35 mM of NaHCO_3_, pH = 9.6 buffer overnight at 4°C. The wells were blocked with TBS-BSA, i.e., 1% BSA in TBS (50 mM of Tris pH 7.4, 150 mM of NaCl) buffer, for 1 h at 37°C, and then washed three times with 0.1% Tween-20 in TBS buffer (TBS-T). Normal human serum (NHS) was collected and pooled from 10 healthy individuals as described above. FD-depleted human serum (ΔFDS) was purchased from Complement Technology, Tyler, TX, USA (cat. no. A336). Sera were diluted six-fold in 10 mM of HEPES, 150 mM of NaCl, 10 mM of EGTA, 4 mM of MgCl_2_, and 0.1% Tween-20, pH = 7.4 buffer. ΔFDS was supplemented with FD at the physiological concentration (0.33 µg/ml) or with pro-FD-R/Q at various concentrations (0.33–100 µg/ml). The diluted serum samples (100 μl) were added to the plate wells and incubated for 20–30 min at 37°C depending on the required sensitivity. The plates were washed 3× with TBS-T and then 5,000-fold-diluted anti-human C3c (Agilent Technologies, Santa Clara, CA, USA, Dako brand, A0062) was added in TBS-BSA. After washing (3× with TBS-T), 40,000-fold-diluted anti-rabbit IgG HRP conjugate (Merck-Sigma-Aldrich, cat. no. A1949) was applied in TBS-BSA. After washing (3× with TBS-T), *O*-phenylenediamine (1 mg/ml) and H_2_O_2_ (0.03%) in 50 mM of citrate pH 5 buffer was used for detection, and H_2_SO_4_ (0.33 M final) was added to stop the reaction. The absorbance was read at 490 nm. The averages of three parallel measurements were normalized to the mean value obtained for NHS or to the mean value obtained for ΔFDS supplemented with FD, as indicated, and provided as percentage values. Statistical analysis was performed by one-way ANOVA. The titration curve was fitted using the OriginPro 8 software by the four parametric logistic function, *y* = *I*_2_ + (*I*_1_ − *I*_2_)/[1 + (*x*/*x*_0_)*^p^*], where *I*_1_ is the minimum intensity, *I*_2_ is the maximum intensity, *x*_0_ is the inflection point (i.e., EC_50_), and *p* is the slope factor.

## Results

### The C3bB complex is a 20 million-fold better substrate than free FB for FD

**Figure 1:**
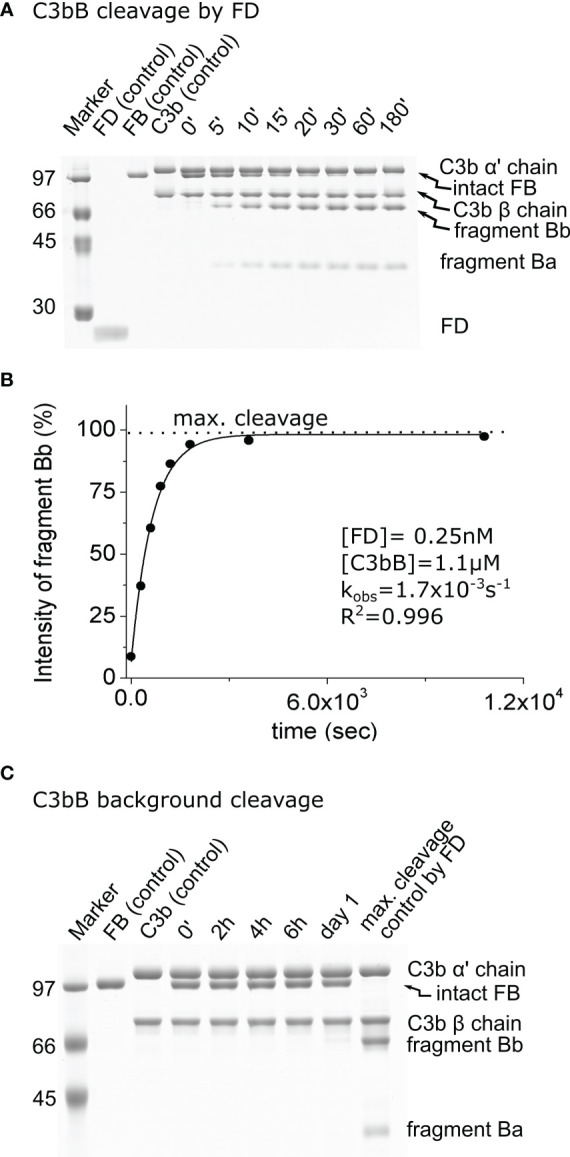
Cleavage of C3bB by factor D (FD). **(A)** The cleavage reaction of C3bB (substrate) by FD was performed at 37°C and monitored by SDS-PAGE under reducing conditions as described in the *Materials and methods*. **(B)** Densitometric analysis was performed to determine the intensities of factor B (FB) cleavage product (Bb) bands. The determined intensities were normalized to the endpoint intensity and plotted as its percentages. The observed rate constant (*k*_obs_) was determined by exponential fit as described in the *Materials and methods*. **(C)** C3bB background cleavage was examined in the absence of an added enzyme. No cleavage was detected within the time interval of the reaction between FD and C3bB (3 h), but a faint band can be detected after 1 day. Densitometric analysis and quantification was also performed to determine the background cleavage *k*_obs_ value (not shown). A typical experiment of each type is presented here. Averages and the number of parallels are listed in **Table 2**.

In order to quantitate the enhancement of the catalytic activity of mature FD on its complex natural substrate, first we measured its activity on C3bB and free FB. We used FB at a 1.1-μM final concentration and C3b at a slight molar excess of 1.3 μM. As expected, FD proved to be a highly efficient enzyme for the cleavage of the C3bB substrate. A 3-h incubation is sufficient to achieve a virtually complete cleavage of C3bB at only 0.25 nM FD concentration (**Figures 1A, B**). The average estimated catalytic efficiency (*k*_obs_/[*E*]_T_) was calculated to be 6.5 × 10^6^ M^−1^ s^−1^ (**Table 2**), indicating a very fast reaction close to the diffusion limit. Note that C3b and FB are only partially associated having a K_D_ of approximately 1.4 μM (38), which gives an approximate 40% saturation of FB with C3b under the assay conditions. Keeping this in mind, the actual catalytic efficiency might be even higher.

**Table 2:** The kinetic parameters of the examined cleavage reactions.

Substrate	Enzyme	[*E*]_T_ (nM)	*k*_obs_ (s^−1^)	*k*_obs_/[*E*]_T_ (M^−1^ s^−1^)	Approx. relative efficiency
C3bB	None (background)	–	(1.0 ± 0.1) × 10^−6a^	–	–
	FD	0.25	(1.6 ± 0.1) × 10^−3^	(6.5 ± 0.5) × 10^6^	1
	pro-FD-R/Q	100	(7.8 ± 0.2) × 10^−4^	(7.8 ± 0.2) × 10^3^	1/800
	MASP-1cf	1,000	(2.3 ± 0.6) × 10^−4^	(2.3 ± 0.6) × 10^2^	1/30 000
	MASP-3cf	2,000^b^	(2.6 ± 1.0) × 10^−6^	∼0.9^c^	negligible
FB	None (background)	–	ND	–	–
	FD	2,000	(6.4 ± 1.4) × 10^−7^	0.3 ± 0.07	1/20,000,000
	pro-FD-R/Q	2,000	(2.3 ± 0.7) × 10^−7d^	∼0.1^c^	negligible
	MASP-1cf	2,600	(6.4 ± 2.3) × 10^−6^	2.5 ± 0.9	1/3,000,000
	MASP-3cf	10,000^b^	ND	–	–

The k_obs_/[E]_T_ value can be considered as an estimate for the catalytic efficiency (k_cat_/K_M_). The average ± SD values were determined from three parallels, except when stated otherwise.

ND, no detectable cleavage.

a The low-level background cleavage was probably due to a trace contaminant in C3b.

b Approximately 90% active and 10% zymogen.

c Close to the detection limit defined by the background. Only an approximate k_obs_/[E]_T_ could be determined.

d Average ± SD from two parallels.

**Figure 2:**
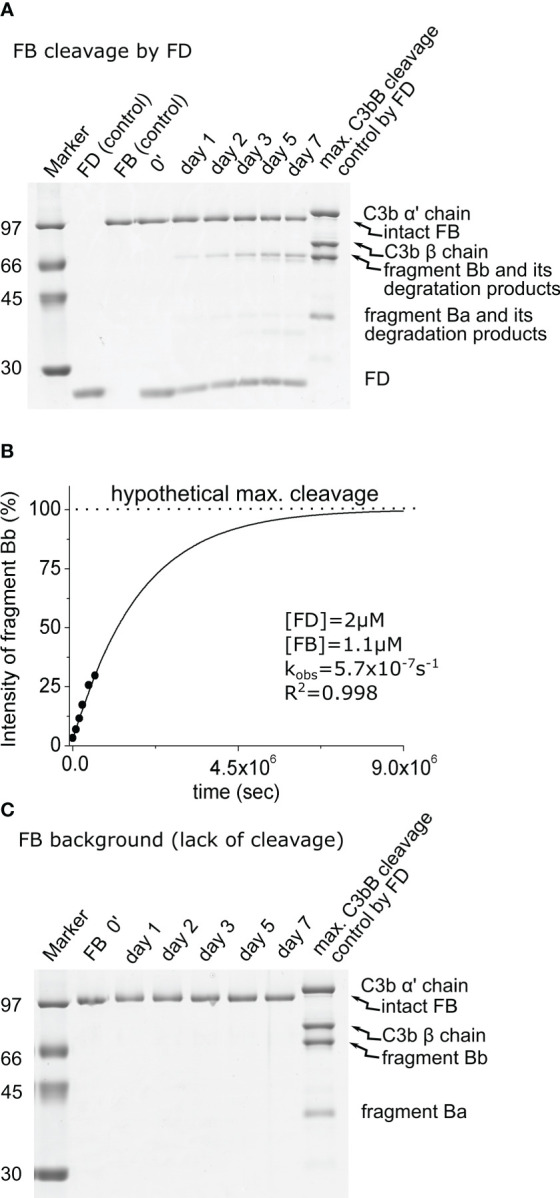
Cleavage of free FB by FD. **(A)** The cleavage reaction of FB (substrate) by FD was performed at 37°C and monitored by SDS-PAGE under reducing conditions as described in the *Materials and methods*. Due to the long incubation time, further degradation of the cleavage products could be observed. Bb was only minimally degraded. **(B)** Densitometric analysis was performed to determine the intensities of the FB cleavage product (Bb) bands. The intensity of degraded Bb was added to the intact Bb intensity for each lane. The hypothetical Bb maximal intensity value was derived from a control reaction between FD and C3bB. The determined intensities were normalized to the value of the maximal cleavage control and plotted as its percentages. The observed rate constant (*k*_obs_) was determined by exponential fit as described in the *Materials and methods*. **(C)** Factor B background cleavage was examined in the absence of the added enzyme. No cleavage was detected during 1 week of incubation. A typical experiment of each type is presented here. Averages and the number of parallels are listed in **Table 2**.

When free FB (1.1 μM) was incubated with 2 μM of FD, a detectable amount of fragment Bb started to appear after 1 day, and the reaction was far not complete even after a week. During this long incubation period, further degradation of Bb and Ba products was observed, which was taken into account during the evaluation of the results (**Figures 2A, B**). We obtained a very low, 0.3 M^−1^ s^−1^, catalytic efficiency (*k*_obs_/[*E*]_T_) toward FB (**Table 2**). This value is physiologically not relevant. The ratio of the catalytic efficiency values shows that the C3bB complex is an approximately 20 million-fold better substrate than FB alone for FD.

### Checking of the background cleavage of C3bB and FB

We also checked the background cleavage of the substrates, without any added enzyme, to trace any potential contaminant interfering with the cleavage reactions. The purity of C3b was over 90% using the improved purification method. Faint contaminant bands are primarily degradation products of C3b. The purity of FB was over 95%. We used FB at 1.1 μM final concentration and C3b at a slight molar excess of 1.3 μM, to compensate for the somewhat lower purity. After 1 day, a low amount of the FB cleavage product, Bb, appeared when the C3bB complex was incubated in the assay buffer (**Figure 1C**). This low background cleavage is probably due to a trace contaminant in the prepared C3b sample. The observed first-order rate constant for the background cleavage (*k*_obs_ = 1.0 × 10^−6^ s^−1^) was taken into consideration as a correction factor for enzymatic reactions involving C3bB (**Table 2**). The background cleavage was negligible compared with the *k*_obs_ values obtained for the FD, pro-FD-R/Q, and MASP-1cf reaction mixtures; however, it was comparable to the value obtained for the reaction involving MASP-3cf, as we will see later.

No cleavage was detected in the mixture containing only FB in the assay buffer in the absence of an added catalyzing enzyme after 1 week of incubation (**Figure 2C**; **Table 2**); therefore, no background cleavage correction was required for the enzymatic reactions involving free FB as the substrate.

### Pro-FD has significant enzymatic activity toward C3bB, modeled by the pro-FD-R/Q variant

In order to model the potential activity of proenzymic FD, we have prepared a stable zymogenic variant by replacing the Arg residue with Gln in the scissile bond. We measured the activity of this variant, pro-FD-R/Q, on C3bB and free FB in a similar fashion as described previously.

**Figure 3:**
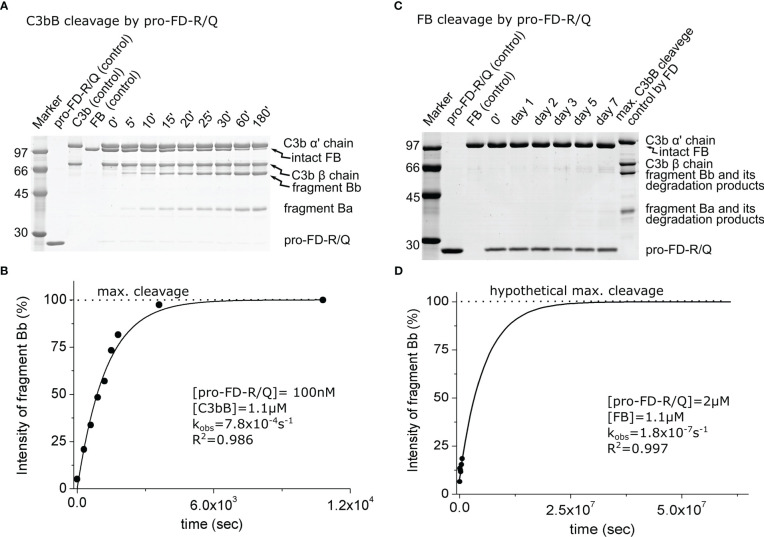
Cleavage of C3bB or FB by pro-FD-R/Q. **(A)** The cleavage reaction of C3bB (substrate) by pro-FD-R/Q was performed at 37°C and monitored by SDS-PAGE under reducing conditions as described in the *Materials and methods*. **(B)** Densitometric analysis was performed to determine the intensities of the FB cleavage product (Bb) bands on the gel in **(A)**. The determined intensities were normalized to the endpoint intensity and plotted as its percentages. The observed rate constant (*k*_obs_) was determined by exponential fit as described in the *Materials and methods*. **(C)** Cleavage of free FB (substrate) by pro-FD-R/Q was performed at 37°C and monitored by SDS-PAGE under reducing conditions as described in the *Materials and methods*. The contrast in this panel is enhanced to make the faint band more visible. **(D)** Densitometric analysis was performed to determine the intensities of the Bb bands on the gel in panel **(C)**. Faint bands probably due to keratin contamination made it difficult to determine reliable intensities. The hypothetical Bb maximal intensity value was derived from a control reaction between FD and C3bB. The determined intensities were normalized to the value of the maximal cleavage control and plotted as its percentages. The observed rate constant (*k*_obs_) was determined by exponential fit as described in the *Materials and methods*. A typical experiment of each type is presented here. Averages and the number of parallels are listed in **Table 2**.

During the cleavage reaction of C3bB by pro-FD-R/Q, almost the entire amount of the FB component was consumed within 3 h, just like in the case of mature FD, although at a higher (100 nM) enzyme concentration (**Figures 3A, B**). The catalytic efficiency (*k*_obs_/[*E*]_T_) of 7.8 × 10^3^ M^−1^ s^−1^ determined for pro-FD-R/Q is relatively high (**Table 2**), and it is only approximately 800-fold lower than the value obtained for mature FD. This value indicates that even the zymogen form of FD can potentially lead to AP C3 convertase production and amplification of the alternative pathway. The potential significance of the activity of pro-FD will be discussed later.

When free FB was used as the substrate, we observed a very low catalytic efficiency for pro-FD-R/Q very close to the detection limit of the method (approx. 0.1 M^−1^ s^−1^) (**Figures 3C, D**; **Table 2**). This very low value is physiologically not relevant, just like in the case of mature FD; however, it is only approximately three-fold lower than the one obtained for mature FD.

In all, our data support the idea that the complex substrate (C3bB) has a major contribution to the enzymatic activity of FD, and the proenzyme to mature enzyme conversion has a much lower impact.

### C3bB and FB cleavage by MASP-1 indicates increased susceptibility of C3b-complexed FB

**Figure 4:**
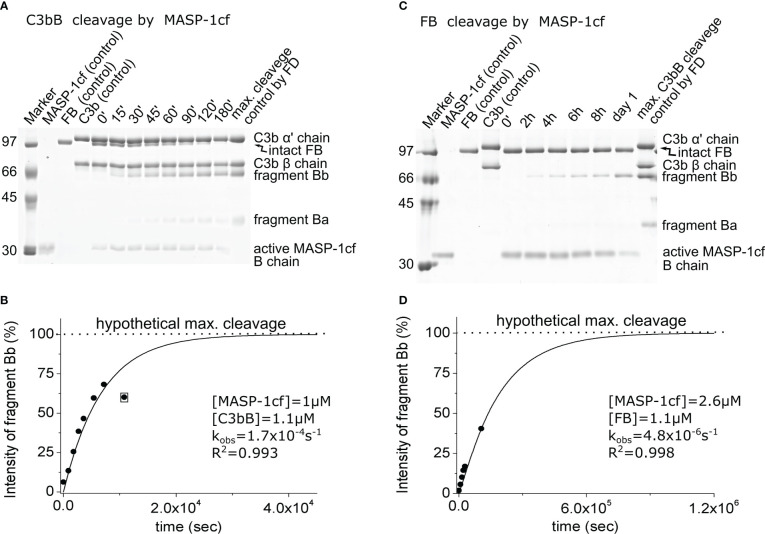
Cleavage of C3bB or FB by MASP-1cf. **(A)** The cleavage reaction of C3bB (substrate) by MASP-1cf was performed at 37°C and monitored by SDS-PAGE under reducing conditions as described in the *Materials and methods*. **(B)** Densitometric analysis was performed to determine the intensities of the FB cleavage product (Bb) bands on the gel in panel **(A)**. The hypothetical Bb maximal intensity value was derived from a control reaction between FD and C3bB. The determined intensities were normalized to the value of the maximal cleavage control and plotted as its percentages. The observed rate constant (*k*_obs_) was determined by exponential fit as described in the *Materials and methods*. An outlier data point, indicated by boxing, was excluded from the data set during the fit. **(C)** Cleavage of free FB (substrate) by MASP-1cf was performed at 37°C and monitored by SDS-PAGE under reducing conditions as described in the *Materials and methods*. A longer examination was not feasible due to the autodegradation of MASP-1cf. **(D)** Densitometric analysis of the gel in panel **(C)** was performed as described for panel **(B)**. A typical experiment of each type is presented here. Averages and the number of parallels are listed in **Table 2**.

Due to its relatively broad substrate specificity, MASP-1 accepts both C3bB and FB as substrates. MASP-1 was included in this study to assess the enhanced susceptibility of FB upon complex formation with C3b, not because of its presumed physiological relevance. In this study, we used the active recombinant catalytic fragment of MASP-1 (MASP-1cf), which can be produced in high purity. MASP-1cf proved to be a much less efficient enzyme on C3bB than FD (**Figures 4A, B**; **Table 2**), and its catalytic efficiency toward C3bB (*k*_obs_/[*E*]_T_ = 230 M^−1^ s^−1^) was found to be approximately 30,000-fold lower than that of FD.

Interestingly, MASP-1cf was about an order of magnitude more efficient on free FB than FD; however, the catalytic efficiency (*k*_obs_/[*E*]_T_ = 2.5 M^−1^ s^−1^) still indicates a slow reaction (**Figures 4C, D**; **Table 2**). What is more important, our results indicate that C3bB is a better substrate (approximately 100-fold) for proteolysis in general, than free FB, which is consistent with previous observations (15) and will be discussed later.

### Lack of C3bB and FB cleavage by MASP-3

**Figure 5:**
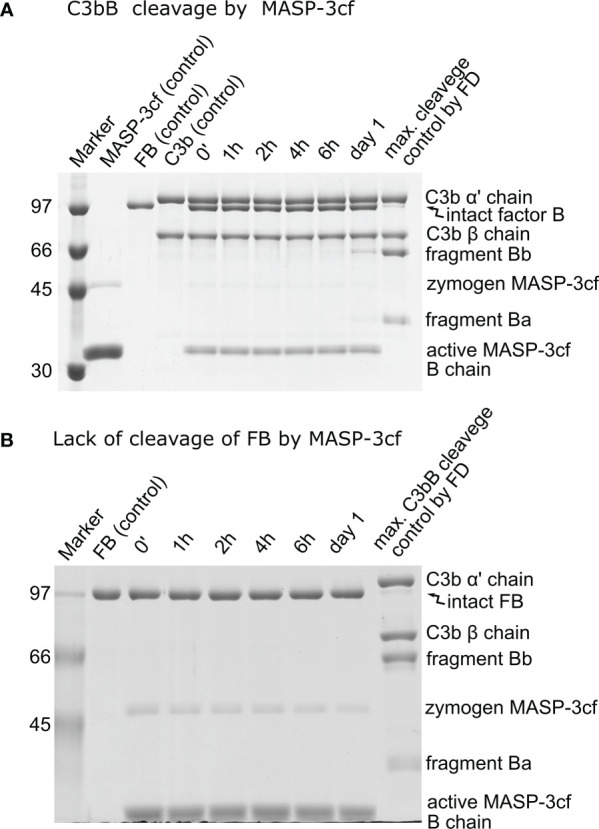
Lack of significant cleavage of C3bB or FB by MASP-3cf. **(A)** The cleavage reaction of C3bB (substrate) by MASP-3cf was performed at 37°C and monitored by SDS-PAGE under reducing conditions as described in the *Materials and methods*. Only a trace amount of the cleavage product fragment Bb appeared after 1 day of incubation. Densitometric analysis and quantification was performed to determine the *k*_obs_ value (not shown) as described in the previous figures. **(B)** Free FB and MASP-3cf were mixed and incubated at 37°C. Samples were analyzed by SDS-PAGE under reducing conditions as described in the *Materials and methods*. Absolutely no cleavage of FB by MASP-3cf was detected during the incubation. A typical experiment of each type is presented here. Averages and the number of parallels are listed in **Table 2**.

It has been described earlier that MASP-3 could directly cleave and activate C3bB (27). Hence, we have included MASP-3 in this study and checked its activity toward C3bB and free FB. The prepared MASP-3cf sample is virtually free from other contaminants; however, there was some remaining zymogen form (~10%) after activation and purification, which produces an additional band on SDS-PAGE (**Figure 5**). On the other hand, zymogen MASP-3cf has no catalytic activity (33), and the lower concentration of the active form was taken into account in the calculations as a correction factor.

When C3bB was used as a substrate, only very marginal cleavage could be detected despite the high (2 μM) MASP-3cf concentration (**Figure 5A**). The observed cleavage rate (*k*_obs_) was only slightly higher than the background cleavage of C3bB, indicating a very low-efficiency reaction (*k*_obs_/[*E*]_T_ < 1 M^−1^ s^−1^ applying background correction) (**Table 2**). In the case of free FB, we used an even higher MASP-3cf concentration (10 μM); however, virtually, no substrate cleavage was detectable (**Figure 5B**).

Our results indicate that it is very unlikely that MASP-3 has any physiological role in FB cleavage, and MASP-3 has no role beyond pro-FD cleavage in the activation of the alternative pathway.

### Pro-FD-R/Q can restore AP activity

**Figure 6:**
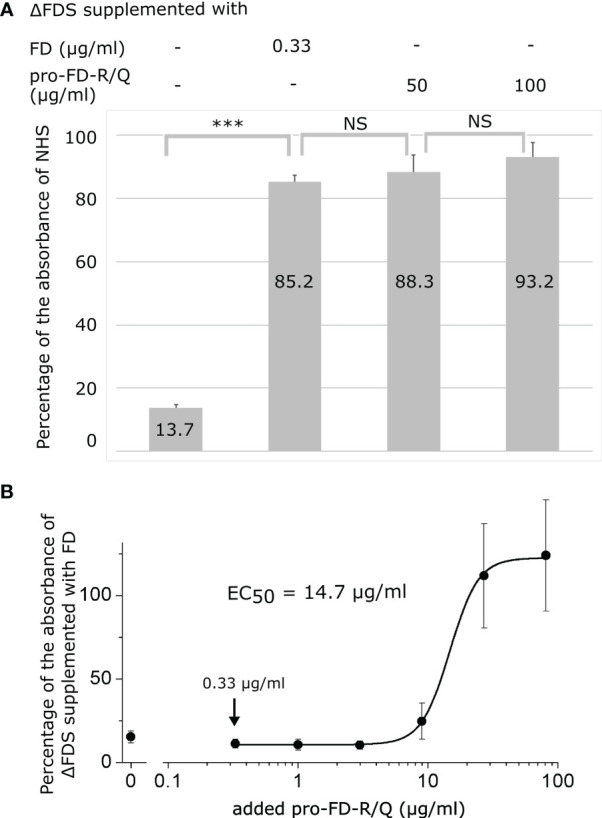
Activity of the alternative pathway (AP) in FD-depleted human serum (ΔFDS) supplemented with mature FD or pro-FD-R/Q. AP activity was measured *via* C3 fragment deposition in six-fold-diluted human serum in zymosan-coated wells. **(A)** Six-fold-diluted ΔFDS was supplemented with mature FD at the physiological concentration (0.33 μg/ml final) or pro-FD-R/Q at high concentrations (50–100 μg/ml). All the supplemented ΔFDS samples had about the same AP activity (85%–93%). Numbers denote percentage values compared with the activity of six-fold-diluted normal human serum (NHS). NS, not significant; *** = highly significant. NHS and ΔFDS were from a different source, which can explain the slight difference between NHS and ΔFDS supplemented with FD. **(B)** Six-fold-diluted ΔFDS was titrated with a three-fold serial dilution of pro-FD-R/Q (0.33 to 81 µg/ml final conc.). The lowest pro-FD-R/Q concentration applied, indicated by an arrow, was equivalent to the physiological FD concentration taking into account the dilution factor. The curve was fitted with the four-parameter logistic function to determine the half-maximal effective concentration (EC_50_). Bars represent SD values. A representative experiment of each type is shown.

AP activity was measured in zymosan-coated wells through the detection of deposited C3 fragments in an ELISA-type assay. First, we have determined the AP activity of ΔFDS compared with NHS. ΔFDS still had a low activity (14%), which was probably due to a trace amount of residual FD in the depleted serum. Supplementing ΔFDS with FD at the physiological concentration has restored the alternative pathway activity to approximately 85%, which is in line with the manufacturer’s specifications. Complementation with 50–100 µg/ml of pro-FD-R/Q resulted in 88%–93% C3 fragment deposition compared with NHS (**Figure 6A**). These activity values do not significantly differ from the activity obtained for ΔFDS supplemented with FD. Recovery of the AP activity suggests that pro-FD, although at a higher concentration, is able to functionally substitute mature FD to initiate the alternative pathway.

Next, ΔFDS was titrated with 0.33–81 µg/ml of pro-FD-R/Q, and the activity was normalized to ΔFDS supplemented with mature FD at the physiological concentration (**Figure 6B**). The lowest pro-FD-R/Q concentration (0.33 µg/ml) is equivalent to the physiological FD concentration at six-fold dilution. A shorter sample incubation time (20 min) was applied to avoid saturation. The EC_50_ of approximately 15 µg/ml suggests that pro-FD-R/Q at approximately 50-fold higher than the physiological FD concentration is enough to restore half of the alternative pathway activity in six-fold-diluted FD-depleted human serum under the assay conditions applied.

## Discussion

In recent years, complement-targeted therapeutics have come of age. A few complement-targeting drugs are in the clinical practice, some are under clinical evaluation, and some are in the preclinical phase (39, 40). A possible approach is to target FD in an attempt to completely inhibit or at least attenuate the AP of the complement system (41–45). When inhibiting FD, though, one must consider the high turnover rate of this small protein (46) and the observation that a relatively low amount of FD was enough to maintain AP activity (47). Another approach is to target MASP-3, which is present mostly as an active enzyme in the blood (48) and serves as the physiological FD maturase enzyme (21, 22, 49). Using a recombinant monoclonal antibody that binds to and inhibits MASP-3, AP inhibition could be attained in mice and cynomolgus monkeys in a few days after dosing due to pro-FD accumulation in the blood (50, 51). During this treatment, FD is filtrated out by the kidneys, and it is replaced by newly synthesized pro-FD in the blood. By sustained inhibition of MASP-3, eventually all factor D will be in the proenzyme form (pro-FD). This method does not give rise to immediate complement inhibition; on the other hand, this approach might have some advantages. AP activity contributing to chronic diseases can be attenuated for a prolonged period; however, the available pool of pro-FD can potentially still provide protection from acute infections (52). The goal of our study was to complete the kinetic landscape of FD and pro-FD with new data. Such data can help interpret future clinical and preclinical results and explain previously published observations.

First, the activity of mature FD was determined on C3bB and free FB. The value obtained for C3bB cleavage (**Table 2**) indicates a very efficient reaction close to the diffusion limit (*k*_obs_/[*E*]_T_ = 6.5 × 10^6^ M^−1^ s^−1^), and it is similar to the previously determined rate constant for C3(H_2_O)B under slightly different conditions (15). The cleavage of FB by FD had been considered to be non-existent and has never been quantified because it is extremely slow (**Table 2**). Indeed, measuring such a low activity (*k*_obs_/[*E*]_T_ = 0.3 M^−1^ s^−1^) is challenging and requires very pure proteins (enzyme and substrate), a high enzyme concentration, and a very long incubation time. For the quantification of this low-efficiency reaction, densitometric analysis of the Bb fragment gave the best results, and because of the incompleteness of the reaction, additional (maximal cleavage) control had to be used for fitting purposes (**Figure 2**).

Our data show that C3bB is approximately a 20 million-fold better substrate for FD than free FB. Using active MASP-1 (catalytic fragment) as a protease with broad specificity, we have found a much lower rate enhancement. Actually, MASP-1 cleaved free FB faster than FD, but on C3bB, it was much less efficient. Nevertheless, the rate enhancement for MASP-1 was approximately 100, which implies that the complex formation with C3b makes FB more susceptible to cleavage in general. This is consistent with the observation that in the crystal structure of FB, the scissile bond is not accessible for cleavage (14), whereas EM studies of the C3bB complex indicated the existence of an “open” and a “closed” conformation, with the “open” conformation comprising of approximately 65% of the pool (12). The rate enhancement observed for FD seems to be a two-stage process, where the conformational change in FB upon complex formation with C3b contributes to a factor of approximately 100, while the substrate-induced conformational change in FD by C3bB might contribute to a further ca. 200,000-fold activity enhancement.

Previously, Taylor and colleagues (15) attempted to model free FB cleavage by using synthetic peptides spanning the FB cleavage site. They could not reliably quantitate the reaction and estimated a *k*_cat_/*K*_M_ value of less than 0.5 M^−1^ s^−1^. Along with the *k*_cat_/*K*_M_ of 2 × 10^6^ M^−1^ s^−1^ they determined for C3(H_2_O)B cleavage, they estimated the rate enhancement to be >10^6^. Our data are consistent with this previous study but provide more accurate numbers.

We also checked if active MASP-3 (catalytic fragment) has any activity on FB or C3bB because it has been implicated that MASP-3 can directly activate C3bB (27). On free FB, no cleavage was detected, whereas C3bB was cleaved at a rate very close to the baseline, despite the high MASP-3 concentration used (**Table 2**). Our data indicate that the previously reported activation of C3bB by MASP-3 was probably just an artifact.

Second, we wanted to address whether pro-FD has any activity. While insect cells produce pro-FD in the cell culture supernatant, traces of active FD are also present (unpublished observation). In order to alleviate this problem and prevent any activation during purification or during the experiments, we mutated the Arg residue of the activation site of pro-FD to Gln, and the resulting mutant variant, pro-FD-R/Q, was used in this study. Pro-FD-R/Q is a stable zymogen, which cannot be activated by a basic residue-specific protease. We determined the catalytic efficiency of pro-FD-R/Q on C3bB. The reaction is easily detectable in a matter of hours, although the concentration of pro-FD-R/Q was higher than the one used for FD (**Table 2**). Still, it was roughly equivalent to the physiological concentration of FD in humans, which is approximately 2 μg/ml or 80 nM (53). The determined catalytic efficiency is quite high (*k*_obs_/[*E*]_T_ = 7.8 × 10^3^ M^−1^ s^−1^), representing a moderately fast proteolytic reaction. On the other hand, it is approximately 800-fold lower than the value obtained for mature FD, indicating that under normal physiological conditions when mature FD is available, this activity is negligible in the blood. On free FB, pro-FD-R/Q had a very low activity similar to the mature enzyme (**Table 2**). The activity of pro-FD might be relevant when the FD maturase (MASP-3) enzyme is missing (e.g., in certain 3MC patients) or when MASP-3-targeted therapy is applied. The activity of pro-FD can explain the observations of Ruseva et al. seen in FH (*FH^−/−^*) and MASP-1/3 (*MASP1^−/−^*) double KO mice (30). Some C3(H_2_O) is continuously produced in the blood, even under normal circumstances, which forms the C3(H_2_O)B pro-convertase complex. This pro-convertase is cleaved by FD (or pro-FD) to form the C3(H_2_O)Bb convertase. In FH-sufficient mice, the fluid-phase C3 convertase rapidly decays preventing excessive activation. However, when FH is missing, uncontrolled complement activation can occur depleting complement components. It appears that the activity of pro-FD in FH (*FH^−/−^*) and MASP-1/3 (*MASP1^−/−^*) double KO mice is sufficient to support the pro-convertase to convertase reaction.

Our results can at least partially explain the residual AP activity observed by Degn et al. in a 3MC patient (28). This patient was homozygous for a nonsense mutation in the common part of the *MASP1* gene resulting in deficiency for both MASP-1 and MASP-3. AP activity was just minimally reduced measured by rabbit erythrocyte lysis assay. One contributing factor for the observed AP activity could be attributed to the proenzymic activity of pro-FD present in the serum of this patient (54, 55). Another contributing factor may be attributed to the use of serum; in complement assays, typically, serum is applied as the source of complement. There are a number of enzymes (e.g., thrombin) that can activate at least a small portion of wild-type pro-FD in the serum or during serum preparation. On the other hand, it has been also described that kallikrein can directly cleave the AP C3 pro-convertase (56). In order to investigate the effect of pro-FD and eliminate the possibility of its activation, we used the stable zymogen pro-FD-R/Q variant, as mentioned before.

Finally, we checked if pro-FD-R/Q was able to restore the activity of the AP in ΔFDS. One must note that the *in vitro* activity of the AP can depend on the assay type. Here, we used six-fold-diluted serum and zymosan as the activator. Adding pro-FD-R/Q at a supraphysiological concentration restored the AP activity to the same level when FD was added at the physiological concentration to the same ΔFDS batch (**Figure 6A**). The half-maximal concentration of pro-FD-R/Q in six-fold-diluted serum was approximately 15 μg/ml, which is approximately 50-fold higher than the physiological concentration of FD, taking into account the dilution factor (**Figure 6B**). Under the assay conditions, no AP activation above the background level was detected at 3 μg/ml of pro-FD-R/Q or less. Note that the background level was not zero, because ΔFDS may have contained traces of FD. Nevertheless, our results demonstrate that pro-FD can potentially substitute for FD in blood samples. Interestingly, in a completely independent recent study, others found a plasma concentration-dependent AP activity in MASP-3-deficient mice, and they also concluded that pro-FD might be able to support complement activation (57).

**Figure 7:**
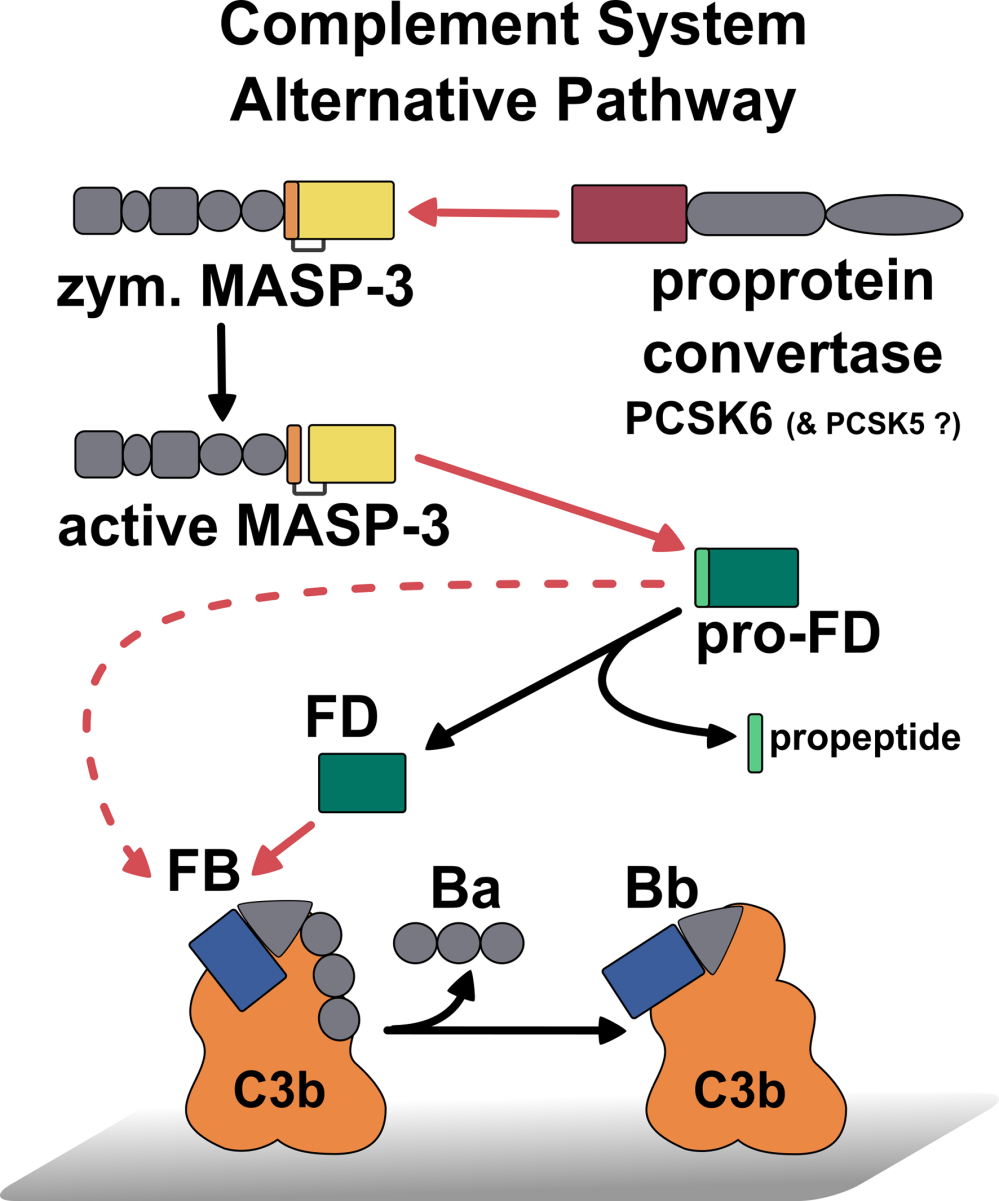
Summary of the first activation steps of the AP of complement, including the recently discovered homeostatic MASP-3 activation (29), the homeostatic activation of pro-FD by MAPS-3 (21), and the low-level activity of pro-FD toward C3bB. Red lines indicate proteolytic cleavage pointing from the enzyme to the substrate, while black lines designate conversion. The cleavage of C3bB by pro-FD is indicated by a dashed red line. This reaction could be relevant in the case of MASP-3 deficiency or MASP-3 inhibition when pro-FD accumulates as the predominant, or even, as the only form in the blood.

The PCSK6–MASP-3–FD axis serves as a homeostatic, preinitiation phase of complement activation (58). MASP-3 is continuously activated by the proprotein convertase PCSK6 in human blood, while MASP-3 cleaves pro-FD to produce the mature enzyme FD. Now, we could complement this picture with the activity of pro-FD (**Figure 7**). MASP-3 and FD are potential therapeutic targets to treat diseases, where complement is a major contributing factor (41, 42, 50, 51). Our results complete the kinetic landscape of FD and pro-FD with new data that can help interpret previous observations and possibly future preclinical experiments and clinical investigations. The measured activity of pro-FD on C3bB might be particularly important. This low-level activity of pro-FD can be relevant in MASP-3-targeted therapies when pro-FD accumulates in the blood during treatment.

## Data availability statement

The original contributions presented in the study are included in the article/supplementary material. Further inquiries can be directed to the corresponding author.

## Ethics statement

The studies involving human participants were reviewed and approved by NNK formerly ÁNTSZ, 1097 Budapest, Albert Flórián út 2-6 (permission number: TUKEB 9190-1/2017/EKU). The patients/participants provided their written informed consent to participate in this study.

## Author contributions

RD, GO, and JD designed the experiments, analyzed the data, and drafted the manuscript. RD prepared the figures. JD made corrections to the figures. RD, GO, RM, BF, BD, and EV carried out the experiments and evaluated the data. PZ and PG corrected the manuscript and assisted in the supervision of the study. JD conceived and supervised the study and wrote the final manuscript. All authors contributed to the article and approved the submitted version.
